# Establishment of a Rapid Detection Technique Based on RPA-LFD and RPA-CRISPR/Cas12a on *Phytophthora pini*

**DOI:** 10.3390/microorganisms13040863

**Published:** 2025-04-10

**Authors:** Tingting Dai, Yufang Guo, Tongyue Wen, Sinong Yu, Yuan Tao, Zhuo Liu

**Affiliations:** 1 Co-Innovation Center for the Sustainable Forestry in Southern China, Nanjing Forestry University, Nanjing 210037, China; 18877359801@163.com (Y.G.); tywen940822@126.com (T.W.); roseandrenard@163.com (Y.T.); 2240100016@njfu.edu.cn (Z.L.); 2 Advanced Analysis and Testing Center, Nanjing Forestry University, Nanjing 210037, China; 3 Modern Forestry Innovation Center of Yancheng State-Owned Forest Farm, Yancheng 224049, China; thq6669@163.com

**Keywords:** *Phytophthora* dieback, recombinase polymerase amplification, lateral flow dipstick, CRISPR/Cas12a, rapid diagnosis

## Abstract

*Phytophthora pini*, a globally dispersed plant pathogen, poses a significant threat to natural ecosystems and cultivated horticultural crops. Early and precise detection of *P. pini* is essential for effective disease management. This study focused on developing specific, rapid, and sensitive molecular diagnostic techniques to identify the pathogenic oomycete *P. pini*. We employed recombinase polymerase amplification with lateral flow device (RPA-LFD) and RPA combined with CRISPR/Cas12a. The RPA-LFD method can identify *P. pini* at concentrations as low as 10 pg/μL in 30 min, while the RPA-CRISPR/Cas12a approach can detect the pathogen at 1 pg/μL in approximately 50 min. These methods are highly effective in identifying disease caused by *P. pini* and provide a basis for future field detection, which may reduce the economic losses associated with this devastating disease.

## 1. Introduction

Oomycetes are a ubiquitous class of filamentous microorganisms that represent a significant threat to global food security and natural ecosystems. Among many phytopathogenic oomycetes, those in the genus *Phytophthora* affect agriculture, horticulture, forestry, and natural ecosystems [1]. First described in 1925, *Phytophthora pini* was later synonymized with *Phytophthora citricola* in 1963 [2,3]. In 2011, *P. pini* was firmly re-established as a separate entity via DNA sequencing and analysis [4]. This phytopathogen has been identified on numerous plants exhibiting symptoms of wilt, dieback, and root rot from locations across North America, Europe, and Asia. At least 18 distinct plant species are affected by *P. pini* in China alone, including *Lonicera hispidula*, *Picea abies*, *Abies fraseri*, and *Rhododendron pulchrum*. Concerns over the introduction and spread of *P. pini* have intensified due to the globalization of trade. Given its well-documented devastating effects on plants, further spread of *P. pini* must be avoided [4,5,6,7,8,9,10,11,12,13,14,15]. For example, *P. pini* in the eastern United States has caused severe damage and mortality to introduced species such as *Fagus sylvatica* [16], and Johson et al. have also emphasized that *Phytophthora* causes severe rot diseases in plants [17]. Rhododendron sales in Oregon, USA, a total of USD 11 million, annually represent 26% of the nation’s production. *Phytophthora cinnamomi*, *P. pini*, and *Phytophthora plurivora* can rapidly cause severe root rot in ≥90% of Rhododendrons. Infestations in the fields and nursery crops in container production areas have resulted in considerable losses [18]. Crown blight and root rot in *Olea europaea* forests in the Veneto region of Italy are also caused by *P. pini*. The threat of *P. pini* to nurseries, natural forests, and global economic development necessitates accurate and rapid detection to control and manage this disease.

Traditional detection methods include isolating the pathogen from diseased plant tissue or trapping it from the soil, followed by identification based on pathogen morphology and DNA sequencing.The identification process is time consuming and requires trained experts, so it is not an optimal diagnostic method. Several molecular assays for *Phytophthora* have been developed that include the use of recombinase polymerase amplification (RPA), which is occasionally combined with CRISPR technology for increased specificity and sensitivity, conventional PCR, nested PCR, real-time quantitative PCR (qPCR), loop-mediated isothermal amplification (LAMP), or enzyme-linked immunosorbent assay (ELISA) [19,20,21,22,23,24,25,26]. At present, PCR is the only molecular detection method for *P. pini* [27]. PCR detection methods are expensive and time-consuming. LAMP technology requires two pairs of primers, with difficult design requirements. Although the ELISA method is widely used in the diagnosis of *Phytophthora* diseases, the antibodies that are used are species specific, and cross-reactions may occur between some types of humus and downy mildew. To meet the demand for rapid and reliable diagnostic methods, two molecular diagnostic tools, RPA with Lateral Flow Detection (RPA-LFD) and RPA-CRISPR/Cas12a, were used in this study to improve *P. pini* detection.

Broad applications for RPA are possible because this technology offers true isothermal amplification. To pair oligonucleotide primers with homologous sequences in double-stranded DNA, the RPA reaction uses an enzyme called recombinase, which first forms a complex with the primers. Next, a single-stranded DNA-binding protein stabilizes the resultant D-loop by attaching itself to the displaced DNA strand. Then, if the target sequence is present, polymerase begins DNA amplification using the primers. The amplifying reaction starts and continues quickly. Thus, from a few copies of the target DNA, highly specific DNA amplification occurs within minutes [28,29]. First, fluorescein isothiocyanate (FITC)-labeled probes hybridize with biotinylated recombinase polymerase amplification (RPA) products to form ternary complexes; these complexes subsequently bind to gold-conjugated anti-FITC antibodies immobilized on the lateral flow device (LFD), generating a visible detection signal through colloidal gold nanoparticle aggregation, generating the principle of LFD [30,31]. Probes are blocked and cannot be extended to create probe-primer dimers. The nfo enzyme recognizes and cuts the abasic site (THF) in the probe only when it has bound to its complementary strand. Cutting the abasic site removes the blocked end of the probe, and the probe can act as a primer, thereby generating a product that can be captured by a lateral flow dipstick. As a result, only the desired amplified fragments are available to bind, cleave, and extend the probe to form a product with the opposing biotin primer, and the product is detected using LFD test strips. The result is more obvious (to the human eye) since the test line and the control line are both colored, indicating a positive outcome. For the purpose of rapid visual detection, RPA and LFD can be used together [32].

Guide RNAs (gRNAs) and particular spacer sequences are used by the CRISPR/Cas system to instruct Cas nucleases to recognize and cleave particular nucleic acid sequences, thereby increasing the specificity and sensitivity of RPA [33,34,35]. With great accuracy and precision, the type II-V CRISPR RNA (CVRNA)-guided endonuclease Cas12a can identify and cleave nucleic acid sequences. Downstream of the protospacer adjacent motif (PAM) sequence (TTTN) by 18–25 nt, the target DNA is under the control of the CRISPR RNA (crRNA). The trans-cleavage activity of nonspecific single-stranded DNA (ssDNA) (containing fluorescent groups and quencher labels) is triggered by the Cas12a/crRNA complex, and a fluorimeter can be used to observe the cleavage products [36]. Nevertheless, the ability of the CRISPR/Cas12a system to identify pathogen-specific nucleic acids may be enhanced [37,38]. We recently created an RPA-CRISPR/Cas12a method that combines RPA detection techniques and CRISPR/Cas12a to address this problem. This method has proven effective in identifying bacteria, fungi, viruses, and other pathogens [37,39,40,41].

Accurate and timely diagnosis of *P. pini* is necessary to manage the disease in an economical and efficient manner. Two rapid detection platforms are presented in this work: RPA integrated with CRISPR/Cas12a and RPA with LFD. These cutting-edge methods provide supplementary choices for the molecular identification of *P. pini* and increase the accuracy and speed of plant disease detection.

## 2. Materials and Methods

### 2.1. DNA Extraction from Isolates

Table S1 includes information on the strains used in this study, including their origins and the quantities that were used. The strains were kept at Nanjing Forestry University’s Forest Pathology Laboratory. A NanoDrop 2000c spectrophotometer (Thermo Fisher Scientific, Waltham, MA, USA) was used to measure the concentration of genomic DNA (gDNA), which was extracted from each isolate using a DNAsecure Plant Kit (Tiangen Biotechnology, Beijing, China). To extract environmental DNA (eDNA) from soil samples, a FastDNA SPIN Kit for soil (MP Bio, Solon, OH, USA) was used, according to the manufacturer’s instructions. Briefly, 400 mg of sample was placed in a 2 mL lysis matrix tube along with 978 mL of phosphate buffer and 122 mL of MT buffer. The lysis tubes were homogenized with a FastPrep FP120 instrument (MP Bio) at speed 6 for 40 s. A NanoDrop 2000c spectrophotometer was used to measure the concentrations of eDNA.

### 2.2. Establishment of RPA-LFD Assay for P. pini

#### 2.2.1. *P. pini*-RPA-LFD Primer Design and Probe Design

To select candidate genes for a *P. pini*-specific RPA-Crispr reaction, the annotated complete CDS sequence of *P. pini* was retrieved (https://www.ncbi.nlm.nih.gov/nucleotide/OP856844.1?report=genbank&log$=nucltop&blast_rank=1&RID=5194ZD34016, accessed on 12 April 2024). To identify target genes that were unique to *P. pini*, we initially retrieved all publicly available *Phytophthora* genome sequences. Then, all the 16,386 gene sequences from *P. pini* were used as the queries against the above genomes (e-value cutoff: 1 × 10^−5^) [27], and genes without hits were treated as unique to *P. pini*. The *Ppini_05588* gene was targeted for gene-specific RPA primers. Primers for *P. pini* were created using a DNA Thermostatic Nucleic Acid Amplification Kit (Leshang, Wuxi, China). The downstream primer was labeled with biotin at the 5′ end, and the design of RPA-LFD primers and probes was completed utilizing the specific sequence *Ppini_05588* [27] (Figure S1). Figure S2 contains a list of primers that were used to amplify Ppini_05588. The probe (GCAGATACGGCTTTGGTGAAGTCCATGACAGAACATACTCTTAAAT) was positioned between the two primers. A carboxyfluorescein moiety (FAM) was added to at the 5′ end of the probe. Tetrahydrofuran (THF) residues were used to modify both the 5′ and 3′ ends, which added at least 30 nucleotides and at least 15 nucleotides, respectively. Furthermore, phosphorylation (C3-space) effectively blocked the 3′ end, and a single base gap 30 bp from the 5′ end was also modified with THF residues.

#### 2.2.2. Establishment of an RPA-LFD Reaction System

**Figure 1:**
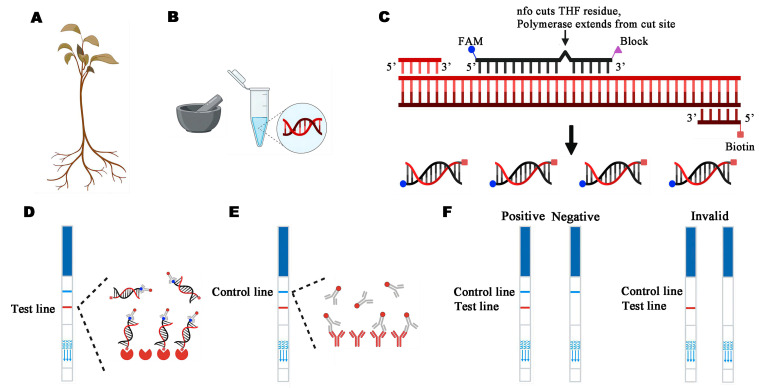
Schematic illustration of the detection of *Phytophthora pini* in actual samples based on Ppini05588-RPA-LFD assays. (**A**) Collection of wild diseased Rhododendron; (**B**) genomic DNA (gDNA) was extracted from samples by using the NaOH method; (**C**) recombinase polymerase amplification (RPA); (**D**) mouse antibody-gold conjugate binds to immobilized anti-mouse IgG antibodies. (**E**) The sample enters the testing line and anti-FAM antibody-AuNPs complex binds to immobilized anti-biotin streptavidin. (**F**) Two bands on the test strip (one blue line for the control and one red line for the test) indicated a positive result. One blue line for the control and no line for the test indicated a negative result. A red line in the control group and none in the test group indicate invalid results.

According to the manufacturer’s instructions, 100 ng/µL of *P. pini* gDNA were used for nucleic acid amplification kit (RAPID method) (Figure 1). The lyophilized enzyme was rehydrated in 25 μL rehydration buffer and 15.2 μL sterile water, and 0.6 μL of 10 μM probe, 2.1 μL of 10 μM reverse primer, and 2.1 μL of 10 μM forward primer were added in that order. Using a hand blender and a quick centrifugation, the mixture was fully dissolved. Next, 3 μL was added to the reaction tube, followed by 2 μL of DNA template and 6 μL of probe (10 μM) in sequential order to reconstitute the lyophilized enzyme powder reaction unit. Then, 2 µL of DNA template was added to the lyophilized enzyme powder following the reaction, and the lyophilized powder was dissolved. After 3 µL of the initiator was added to the reaction tube, it was centrifuged and placed in a metal bath for 4 min at 39 °C. When the tubes were removed from the metal bath, they were centrifuged again. The initial 4 min reaction was confidently continued for an additional 16 min in the metal bath, followed by a centrifugation. After amplification, the product should be diluted 10–100 times with PBS or PBST to ensure accurate results. We used LFD test strips to observe the result. Two bands on the test strip (one blue line for the control and one red line for the test) indicated a positive result. One blue line for the control and no line for the test indicated a negative result.

### 2.3. RPA-CRISPR/Cas12a Primer and crRNA and Optimization of Reaction Conditions

#### 2.3.1. RPA-CRISPR/Cas12a Primer Design and crRNA Design

For the RPA-CRISPR/Cas12a experiment, we chose the specific primer targeting *Ppini05588* based on the preliminary screening results from the RPA-LFD assay. To precisely target this sequence, we used the crRNA for *P. pini*, and the reverse primer was designed without a biotin label at the 5′ end. The CHOPCHOP web application (http://chopchop.org, accessed on 27 May 2024) was used to create the crRNA. The conserved region within the RPA amplicon must be targeted by the crRNA to prevent sequence overlap with the RPA primers and to ensure successful amplification and detection using the RPA method (Figure S1). The ssDNA reporter (5′ 6-FAM-TTATT-BHQ), which is labeled with 6-FAM at the 5′ end and BHQ-1 at the 3′ end, requires protection from light. The ssDNA reporter, crRNA, and RPA primers were all produced by GenScript (Nanjing, China) and were stored at −80 °C until required.

#### 2.3.2. Optimization of RPA-CRISPR/Cas12a Reaction Conditions

**Figure 2:**
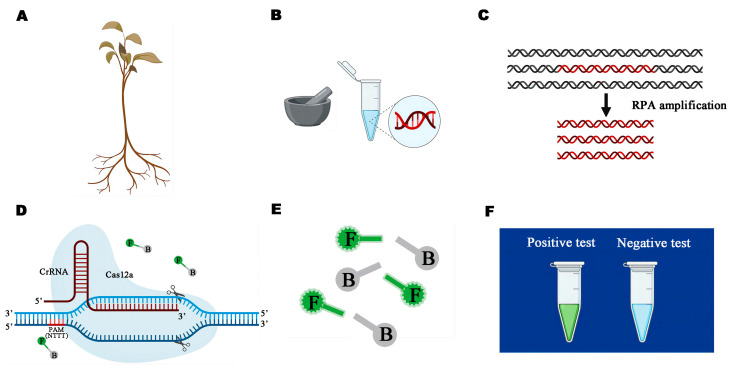
Schematic illustration of the detection of *Phytophthora pini* in actual samples based on Ppini05588-RPA-CRISPR/Cas12a assays. (**A**) Collection of wild diseased Rhododendron. (**B**) Genomic DNA (gDNA) was extracted from samples by using the NaOH method. (**C**) Recombinase polymerase amplification (RPA). (**D**) The semi closed R-ring formed by Cas12a and PAM. (**E**) Binding of CrRNA to Cas12a activates the cleavage activity of Cas12a, which cuts off the incorporated ssDNA reporter containing both the fluorescent moiety FAM and the quenching moiety BHQ, which emits green fluorescence under specific excitation light. (**F**) Detection of visible green fluorescence under a Blue LED Transilluminator at a wavelength of 470 nm.

The RPA reaction and CRISPR/Cas12a-based detection represented the two stages of the RPA-CRISPR/Cas12a assay (Figure 2). Following the manufacturer’s instructions, a test strip kit (Magigen, Guangzhou, China) was used for the RPA assay. A reaction mixture totaling 47 µL included 16 µL of double-distilled water (ddH_2_O), 25 µL of buffer, 2 µL each of forward and reverse primers (Pr061RPA-F/Pr061RPA-R, 10 µM), and 2 µL of gDNA (100 ng/µL). Each reaction required 50 µL of the mixture. The reaction device lid was covered with 3 µL of the supplied activator after the mixture was centrifuged at 4000 rpm for 5 s. To ensure the activator and mixture were properly mixed, the reaction device was securely covered before shaking it by hand for 3 s. Then, the reaction was centrifuged again for 5 s at 4000 rpm 37 °C. The reaction tubes were manually shaken and centrifuged for 5 s at 4000 rpm after 4 min. The CRISPR/Cas12a system was used to determine the RPA product.

In the reaction tube, the ssDNA reporter was introduced by the CRISPR/Cas12a system. The ssDNA reporter was cleaved and fluoresced following recognition by Cas12a/crRNA. A range of crRNA (40 nM, 300 nM, 500 nM, 1 µM, 2 µM, 5 µM, and 10 µM) and ssDNA reporter (40 nM, 300 nM, 1 µM, 2 µM, 5 µM, and 10 µM) concentrations were tested to identify the ideal combination of concentrations (Table S2). Eight time points from 5 to 40 min were assessed to establish the optimal incubation period for the RPA reaction and for Cas12a cleavage. The CRISPR/Cas12a reaction occurred in a 50 µL reaction mix that included 2 µL of RPA product, 1 µL of ssDNA reporter, 3 µL of crRNA, 5 µL of reaction buffer, and 1 µL of deionized water. After centrifugation for 5 s at 4000 rpm, the mixture was stirred and incubated at 37 °C for an additional 5 s. Two approaches were used to assess the RPA-CRISPR/Cas12a test. A Multi-Mode Microplate Reader (Vatioskan Flash, Thermo, Nanjing, China) with an excitation wavelength of 485 nm and an emission wavelength of 520 nm identified distinct fluorescent signals. At 470 nm, green fluorescence was detected under the blue LED transilluminator (Baisai Ltd., Shanghai, China). The STDEVP function was used to analyze the results obtained by repeating CRISPR /Cas12a analysis three times to calculate the standard deviation. Statistical analysis was performed using GraphPad Prism 8 software (GraphPad Software Inc., San Diego, CA, USA). The experimental group and control group were compared via one-way analysis of variance (ANOVA), and *p* < 0.05 (*) was considered significant.

### 2.4. Specificity Validation of the RPA-LFD and RPA-CRISPR/Cas12a Reaction Systems

To evaluate the specificity of the RPA-LFD and CRISPR/Cas12a assays in identifying *P. pini*, 100 ng/μL of gDNA from the *P. pini* was used. Furthermore, RPA-LFD and RPA-CRISPR/Cas12a specific assays employed gDNA templates (Table S1). A positive control (100 ng/μL *P. pini* gDNA) and a negative control (ddH_2_O) were included in each experimental set, which underwent three tests. The data were processed as described above. The experimental group and control group were compared via one-way analysis of variance (ANOVA), and *p* < 0.05 (*) was considered significant.

### 2.5. Sensitivity Validation of the RPA-LFD and RPA-CRISPR/Cas12a Reaction Systems

Dilutions ranging from 100 ng/μL to 100 fg/μL were used for validation. Serially diluted *P. pini* gDNA was used for the sensitivity analysis of the RPA-LFD system, which made use of *P. pini*-specific RPA-LFD primer sets. Similarly, the same dilutions of *P. pini* gDNA were used as templates to validate the sensitivity of the RPA-CRISPR/Cas12a assay. A blue LED transilluminator was used to observe fluorescence at 470 nm, and full-wavelength zymography was used to measure fluorescence intensity. Three repetitions of each set of experiments were conducted, with a positive control (100 ng/μL *P. pini* gDNA) and a negative control (ddH_2_O). The data were processed as described above. The experimental group and control group were compared via one-way analysis of variance (ANOVA), and *p* < 0.05 (*) was considered significant.

### 2.6. Validation of the Two Reaction Systems via Artificially Inoculated Leaves

Young, healthy Rhododendron leaves were selected and sterilized for inoculation. Three times, for a total of 10–60 s, a leaf surface was soaked in 70% ethanol before being rinsed with sterile water. The leaves were then lightly wounded with a sterile inoculation needle. After three days of waiting for *P. pini* to grow in V8 medium, a sterile 6 mm diameter perforator was used to obtain pieces of *P. pini* and V8 medium. Sterile V8 medium was used as a control, and pieces with mycelial surfaces were applied to leaf wounds. Leaf samples for inoculation were then covered with sterile filter paper that had been moistened with sterile water. The petioles were covered with clear cling film and damp cotton for moisture retention. Following incubation at 25 °C (12 h of alternating light and dark), the pathogenesis was noted. The leaves were cleaned in a sterile environment using sterile water. Using NaOH [42] fast lysis, mycelial DNA was extracted from plant tissues. Genomic DNA was extracted from the *R. pulchrum* leaves to serve as a template for RPA. A purified *P. pini* isolate (100 ng/µL) was used as a positive control, and ddH_2_O was used as a negative control for Days 1–5 of inoculation. A positive control used *P. pini* gDNA (100 ng/μL). The presence of two bands on the test strip confirmed the presence of *P. pini* in the sample, while the presence of only one blue band in the quality control area of the test strip indicated a negative result, i.e., *P. pini* was absent from the sample. The RPA-CRISPR/Cas12a assay was performed using gDNA from Rhododendron leaves tissues as a model and *P. pini* gDNA (100 ng/μL) as a template. The positive control reaction also used *P. pini* gDNA (100 ng/μL) as a template. Green fluorescence was observed at 470 nm with a blue LED transilluminator, and fluorescence intensity was measured with full-wavelength zymography. Each experiment included a negative and a positive control and each series of experiments was repeated three times. The data were processed as described above.

### 2.7. Detecting P. pini from Leaves Samples Using RPA-LFD and RPA-CRISPR/Cas12a Assay

A previous study conducted by our laboratory confirmed the presence of *Phytophthora pini* in all 20 samples of Rhododendron plants collected from three parks and gardens in Nanjing, China [15]. DNA from 15 of these plant samples was used for PCR, RPA-LFD and RPA-CRISPR/Cas12a detection. Each DNA sample was tested three times in each assay. Purified gDNA (100 ng) of the *P. pini* isolate was used as a positive control, while DNA from healthy Rhododendron leaves served as a negative control.

## 3. Results

### 3.1. Optimization of Ppini05588-CrRNA and the ssDNA Reporter Based on the Ppini05588-RPA-CRISPR/Cas12a Detection System

**Figure 3:**
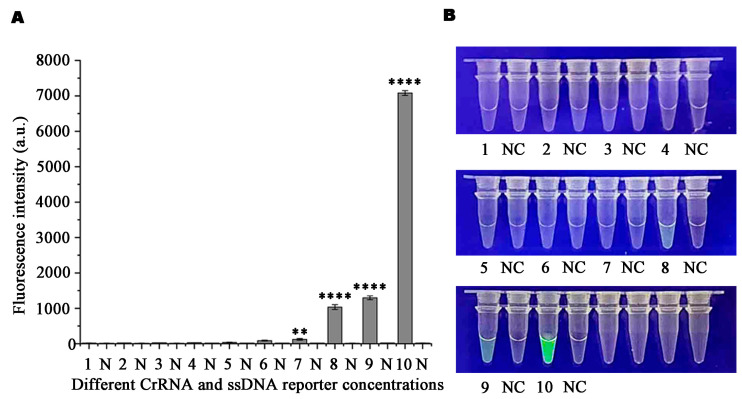
Optimization of the RPA-CRISPR/Cas12a assay crRNA and ssDNA reporter concentrations. (**A**) The concentrations of the crRNA and the ssDNA reporter were set as follows: 1: 40 nM CrRNA, 40 nM ssDNA reporter; 2: 300 nM CrRNA, 40 nM ssDNA reporter, 3: 500 nM CrRNA, 300 nM ssDNA reporter, 4: 1 µM CrRNA, 1 µM ssDNA reporter, 5: 1 µM CrRNA, 2 µM ssDNA reporter, 6: 2 µM CrRNA, 1 µM ssDNA reporter, 7: 5 µM CrRNA, 1 µM ssDNA reporter, 8: 1 µM CrRNA, 5 µM ssDNA reporter, 9: 10 µM CrRNA, 5 µM ssDNA reporter, 10: 10 µM CrRNA, 10 µM ssDNA reporter, NC: Negative control (double-distilled H_2_O). Fluorescence detection was performed using a multi-functional microplate reader (λex: 485 nm; λem: 520 nm); (**B**) Detection of green fluorescence in the visible range under a blue LED transilluminator at a wavelength of 470 nm. *p* < 0.01 (**), *p* < 0.0001 (****).

**Figure 4:**
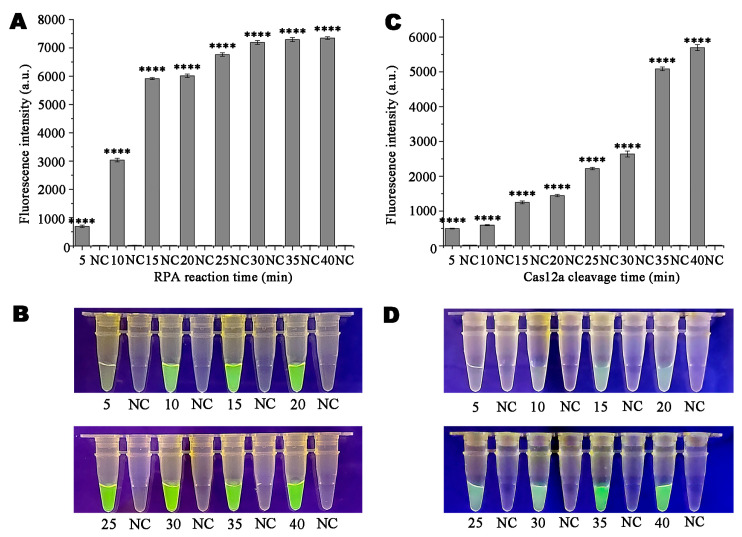
Optimization of recombinase polymerase amplification (RPA) reaction time and Cas12a cleavage time for the RPA-CRISPR/Cas12a assay. (**A**,**C**) Detection of relative fluorescence intensity by a multifunctional microplate reader (λex: 485 nm; λem: 520 nm). The standard deviation is shown in the figure as an error bar. (**B**,**D**) Detection of visible green fluorescence under a Blue LED Transilluminator at a wavelength of 470 nm. (**A**,**B**) RPA reaction times (min): 5, 10, 15, 20, 25, 30, 35, 40 min. NC: negative control. (**C**,**D**) Cas12a cleavage times (min): 5, 10, 15, 20, 25, 30, 35, 40 min. NC: negative control. *p* < 0.0001 (****).

To determine the most effective detection concentration via the Ppini05588-RPA-CRISPR/Cas12a reaction system, various concentrations of Ppini05588-CrRNA probe and ssDNA reporter were used. The fluorescence intensity peaked at concentrations of 1 µM for Ppini05588-CrRNA and 10 µM for the ssDNA reporter, as shown in Figure 3. The specific concentration combinations used are shown in Table S2. Additionally, the reaction times for RPA and CRISPR-Cas12a were investigated to shorten the assay duration. Green fluorescence was observed after 5 min of exposure to the blue LED transilluminator at 470 nm during the RPA reaction (Figure 4A,C). However, the clearest and most stable fluorescence was observed after 15 min (Figure 4B,D). Therefore, based on the relative fluorescence intensity via full-wavelength zymography, the optimal duration for the RPA reaction was 15 min. Cas12a cutting was detected after 5 min, when green fluorescence became visible. The fluorescence subsequently strengthened and stabilized after 35 min, which was determined via full-wavelength zymography to be ideal for Cas12a cutting.

### 3.2. Comparative Validation of P. pini Specificity Using the RPA-LFD and RPA-CRISPR/Cas12a Reaction Systems

**Figure 5:**
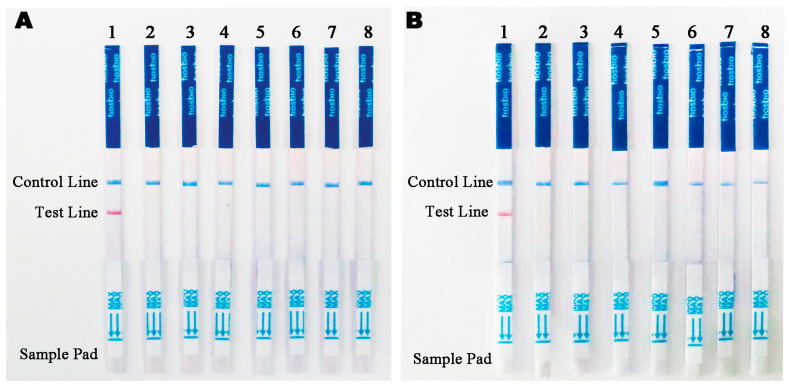
Evaluation of the specificity of the RPA-LFD assay. (**A**) Evaluation using genomic DNA isolated from 1. *Phytophthora pini*. 2. *Phytophthora capsici*. 3. *Phytophthora ramorum*. 4. *Phytophthora palmivora*. 5. *Phytophthora citrophthora*. 6. *Phytophthora litchii*. 7. *Phytophthora megasperma*. 8. Negative control. (**B**) Evaluation using genomic DNA isolated from 1. *Phytophthora pini*. 2. *Phytopythium. litorale*. 3. *Fusarium circinatum*. 4. *Colletotrichum truncatum*. 5. *Alternaria alternata*. 6. *Botryosphaeria dothidea*. 7. *Bursaphelenchus xylophillus*. 8. Negative control. Control Line: Control line. Test Line: Test line.

The DNA of various species, including 32 *Phytophthora* species, 2 *Phytopythium* species, 3 *Pythiaceae* species, 34 fungal species, and 2 *Bursaphelenchus* species (Table S1), was amplified using the Ppini05588-RPA-F upstream primer and the Ppini05588-RPA-R downstream primer at 100 ng/µL Only *P. pini* DNA caused a red band on the test strip and a blue band on the control area. Test strips for the remaining oomycetes, fungus, nematode, and the negative control strips displayed a blue band in the quality control (QC) strip. It is also important to note that no red band appeared on the control area of the strip. No red band appeared on the test strip, and a blue band appeared only on the control line; there was no red band on the test line or the control line of the test strip (Figure 5A,B).

**Figure 6:**
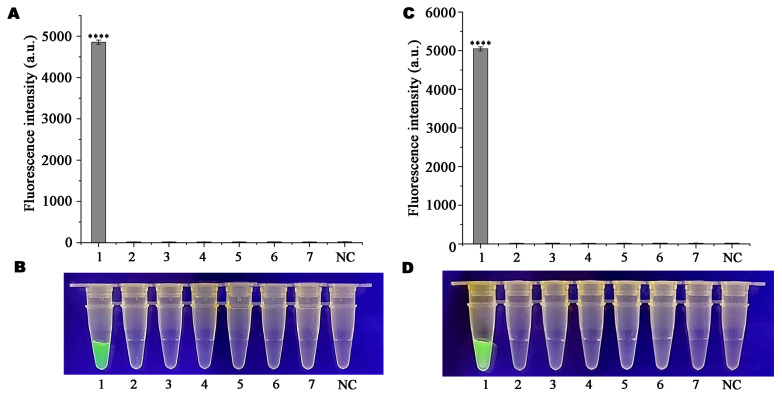
Specificity evaluation of *Phytophthora pini* based on *Ppini05588*-RPA-CRISPR/Cas12a assays. (**A**,**C**) Detection of relative fluorescence intensity by a multifunctional microplate reader (λex: 485 nm, λem: 520 nm). The standard deviation is shown in the figure as an error bar. (**B**,**D**) Detection of visible green fluorescence under a Blue LED Transilluminator at a wavelength of 470 nm. (**A**,**B**) 1. *Phytophthora pini*. 2. *Phytophthora capsici*. 3. *Phytophthora ramorum*. 4. *Phytophthora palmivora*. 5. *Phytophthora citrophthora*. 6. *Phytophthora litchii*. 7. *Phytophthora megasperma*. NC. Negative control. (**C**,**D**) 1. *Phytophthora pini*. 2. *Phytopythium litorale*. 3. *Fusarium circinatum*. 4. *Colletotrichum truncatum*. 5. *Alternaria alternata*. 6. *Botryosphaeria dothidea*. 7. *Bursaphelenchus xylophillus*. NC. Negative control. *p* < 0.0001 (****).

RPA was performed using DNA from the same strain as the template to confirm the accuracy of the Ppini05588-RPA-CRISPR/Cas12a-based detection system. Only *P. pini* generated visible green fluorescence at 470 nm when used as a template for the RPA reaction (Figure 6B,D) or produced high relative fluorescence intensity in full-wavelength zymography (Figure 6A,C). Other strains and the negative control (ddH_2_O) did not exhibit any green fluorescence or relative fluorescence intensity or only displayed very low levels.

### 3.3. Comparative Validation of Sensitivity to P. pini Using the RPA-LFD and RPA-CRISPR/Cas12a Reaction Systems

**Figure 7:**
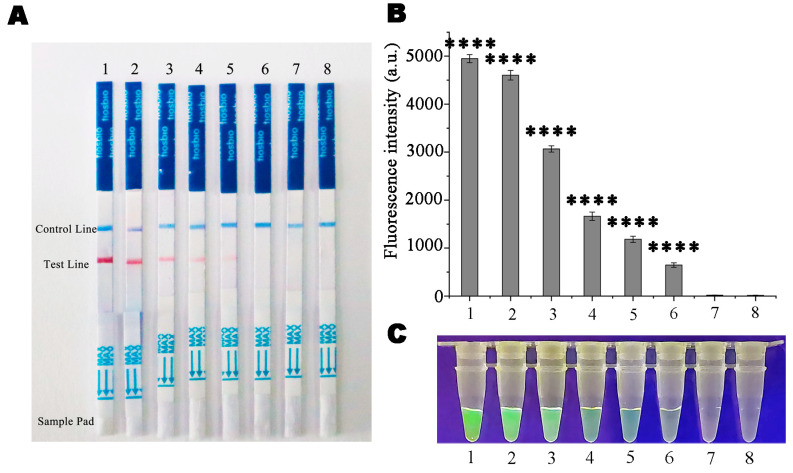
Evaluation of the sensitivity of the RPA-LFD and RPA-CRISPR/Cas12a assay. (**A**) Sensitivity of *Ppini05588*-RPA-LFD test for *Phytophthora pini*. 1. 100 ng/μL. 2. 10 ng/μL. 3. 1 ng/μL. 4. 100 pg/μL. 5. 10 pg/μL. 6. 1 pg/μL. 7. 100 fg/μL. 8. Negative control. Control Line: Control line. Test Line: Test line. Sample Pad: Sample pad. (**B**,**C**) Sensitivity evaluation of *P. pini* based on *Ppini05588*-RPA-CRISPR/Cas12a assays. 1–7: 100 ng/μL, 10 ng/μL, 1 ng/μL, 100 pg/μL, 10 pg/μL, 1 pg/μL, and 100 fg/μL. 8: Negative control. *p* < 0.0001 (****).

To validate the sensitivity of the RPA-LFD and RPA-CRISPR/Cas12a methods, the *P. pini* DNA concentration was gradient diluted 10-fold from 100 ng/µL to 100 fg/µL. The RPA-LFD assay was successful at 10 pg (Figure 7A). The RPA-CRISPR/Cas12a assay demonstrated sensitivity at 1 pg/µL (Figure 7B,C).

### 3.4. Evaluation Results of RPA-LFD and RPA-CRISPR/Cas12a Detection Techniques for Artificially Inoculated Samples

**Figure 8:**
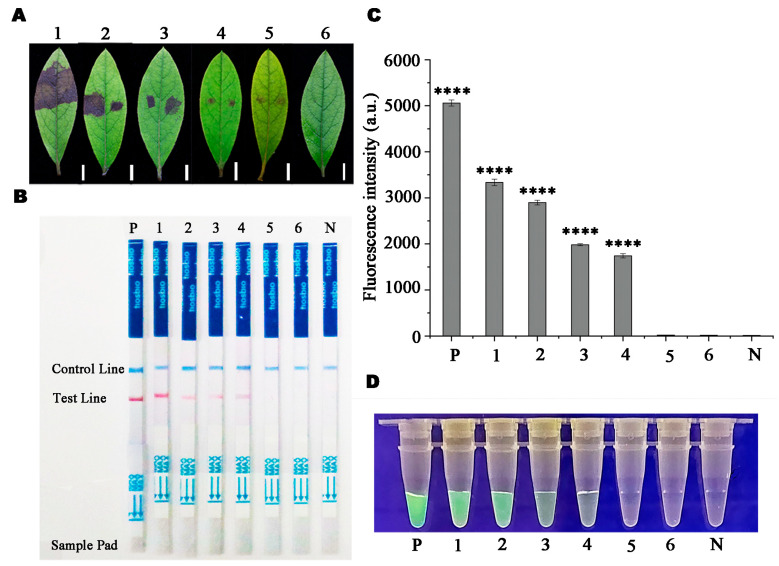
Detection of *Phytophthora pini* in artificially inoculated Rhododendron based on RPA-LFD and RPA-CRISPR/Cas12a assays. (**A**) 1. Symptoms of Rhododendron after five days after inoculation with *Phytophthora pini*. 2. Symptoms of Rhododendron after four days after inoculation with *P. pini*. 3. Symptoms of Rhododendron after three days after inoculation with *Phytophthora pini*. 4. Symptoms of Rhododendron after two days after inoculation with *Phytophthora pini*. 5. Symptoms of Rhododendron after one day of inoculation with *Phytophthora. pini*. 6. Negetive control. Scale bar = 10 mm. (**B**) P: Positive control (*Phytophthora pini* gDNA). 1–5. Detection results for gDNA extracted from inoculated Rhododendron leaves after five, four, three, two days and one day, respectively. 6. Detection results for gDNA extracted from non-inoculated leaves. N: Negative control. Control Line: Control line. Test Line: Test line. Sample Pad: Sample pad. (**C**,**D**) P: Positive control (*P. pini* gDNA). 1–5. Detection results for gDNA extracted from inoculated Rhododendron leaves after five, four, three, two days and one day, respectively. 6. Detection results for gDNA extracted from non-inoculated leaves. N: Negative control. *p* < 0.0001 (****).

One day after inoculation with the *P. pini* isolate, black lesions were observed at the inoculation sites on Rhododendron leaves. By observing the inoculated leaves every day after inoculation, the area of these spots increased, while *R. pulchrum* leaves that were not inoculated with *P. pini* remained healthy (Figure 8A). A test line was detected on Days 2–5 following *P. pini* inoculation, along with positive control DNA. However, no test line was observed on Day 1 for Rhododendron leaves inoculated with *P. pini*, uninoculated samples, and NTC (Figure 8B). In addition, green fluorescence was observed using the RPA-CRISPR/Cas12a reaction system on Day 2, except for the DNA of the positive control. No green fluorescence was observed in leaves from Day 1, gDNA from control leaves, or the negative control (Figure 8C,D).

### 3.5. Comparative Assessment of Detection Methods Using Leaf Samples

RPA-LFD and RPA-CRISPR/Cas12a assays were used for 15 samples of *P. pini* infested plants. The assay results were consistent across three replicates of the experiment. Only 9 of the 15 samples were detected to contain *P. pini* using PCR. *P. pini* was detected in all 15 samples using the RPA-LFD and RPA-CRISPR/Cas12a assays, whereas *P. pini* was not detected in healthy azalea leaf plant samples (Table 1).

## 4. Discussion

*Phytophthora pini* is a soil-borne pathogen that causes root-cap rot in at least 18 plant species from 12 genera in North America, Europe, and Asia [43]. At present, there are few molecular methods for detecting *P. pini*. To implement timely disease management and avoid the costs of misdiagnosis or delayed diagnosis, accurate detection is a prerequisite for the management and elimination of *P. pini*. A specific, reliable method of detecting this pathogen is of great value in improving the diagnosis and management of Phytophthora rot.

In this study, two detection systems for *P. pini*, RPA-LFD and RPA-CRISPR/Cas12a, were successfully established. The RPA-LFD method can be completed in about 30 min, while the RPA-CRISPR/Cas12a detection method takes about 50 min. The RPA-LFD method is notably more time-efficient than the RPA-CRISPR/Cas12a method. Both methods of detecting *P. pini* were highly specific. As for sensitivity, the lowest concentration detectable using RPA-LFD was 10 pg/μL, whereas RPA-CRISPR/Cas12a could detect *P. pini* at 1 pg/μL. Nonetheless, both detection techniques were capable of highly sensitive *P. pini* detection. Inoculating Rhododendron leaves showed that both methods could detect *P. pini* on the second day after inoculation, which demonstrated the accuracy and practicality of both detection methods. *P. pini* was also successfully detected in field plant samples. *P. pini* was also successfully detected in field plant samples by both assays with higher success rates than the PCR assay.

There are no other known detection techniques for *P. pini* except for PCR techniques. The RPA-LFD and RPA-CRISPR/Cas12a methods presented in this study have several benefits over conventional PCR methods. PCR methods require at least 2.5 h, which includes 90 min for PCR amplification and 30 min for gel electrophoresis. Amplification can be performed without a PCR instrument when using the RPA-LFD and RPA-CRISPR/Cas12a assays, and the time to completion is much shorter [44]. Portable qPCR instruments, are useful in the field, but they require high-capacity batteries or automobile batteries that provide high temperatures and thermocycling temperatures. Similarly, LAMP is a highly effective isothermal nucleic acid technology that is frequently used to identify plant pathogens. It is a quick, simple, and portable way to detect pathogens. Unfortunately, its use is limited by its high reaction temperature (60–70 °C), complex primer design (which is more sensitive than PCR [27]), shorter incubation time, and requirement for high-performance batteries. In contrast, RPA-LFD and RPA-CRISPR/Cas12a technologies can operate at 37 °C and yield clear results that do not require complex result interpretation tools.

However, creating distinct, targeted probes can be an expensive undertaking when utilizing RPA-LFD. It is noteworthy that the use of a universal ssDNA reporter tagged with FAM and BHQ-1 (5′ 6-FAM-TTATT-BHQ-1 3′) lowers the cost in RPA-CRIPR/Cas12a technology while attaining high pathogen detection throughput [26,45,46]. While both RPA-CRISPR/Cas12a and RPA-LFD are highly specific detection methods, the combination of RPA and CRISPR/Cas12a provides dual specificity. However, the RPA-LFD method has a non-negligible inherent lack of primer-dependent artifacts. In the RPA-based LFD reaction, forward and reverse primers are labeled at the 5′-end with FAM and biotin, respectively. Amplicons are labeled with biotin and FAM. When primer dimers form, a positive signal is produced regardless of the degree of caution exercised in the screening of primers, and it is virtually impossible to prevent the formation of primer dimers during the process of DNA amplification [43,47,48]. By combining RPA with CRISPR-Cas12a, we are able to detect the target twice, including the recognition of the RPA primer during the RPA reaction and subsequent recognition of the RPA product by CRISPR-Cas12a. This effectively avoided false positives during RPA, which is a limitation of RPA-LFD.

Despite the advantage of RPA-CRISPR/Cas12a over RPA-LFD in terms of reducing the likelihood of false positives, there are still some challenges. For instance, the currently identified Cas12a has limited genomic targeting coverage and editing efficiency, and the cleavage activity of Cas12a relies on the activity of the Cas12a-crRNA complex [49,50,51,52,53]. Additionally, the reagents required for the CRISPR reaction system need to be lyophilized before use, which limits the practicality of RPA-CRISPR/Cas12a in field settings [54,55]. In contrast, RPA-LFD has fewer limitations and is more convenient, making it a more practical option in certain scenarios. In contrast to RPA-CRISPR/Cas12a technology, RPA-LFD technology has been successfully applied in field detection of some pathogens; for example, RPA-LFD technology is currently used to detect harmful pathogens such as *Fusarium oxysporum* and *Bursaphelenchus xylophilus* in the field. Its results are highly accurate and have the same sensitivity as PCR detection, making it a promising tool for portable nucleic acid detection [43,56,57].

## 5. Conclusions

The development of rapid detection techniques, such as RPA-LFD and RPA-CRISPR/Cas12a, is a crucial milestone in plant pathology. These methods offer a fast, specific, and sensitive means of identifying *Phytophthora pini*, a destructive invasive species that causes disease in conifers and other woody plants. RPA-LFD amplifies target DNA sequences under isothermal conditions and detects them using a lateral flow dipstick, providing results within minutes. The integration of the CRISPR/Cas12a system with RPA further enhances the specificity and sensitivity of the detection method. The Cas12a protein, guided by a specifically designed single guide RNA, can cleave non-target DNA and generate a signal that confirms the presence of the target pathogen. This combination leverages the benefits of both isothermal amplification and the highly precise genome editing capabilities of the CRISPR system. Together, these techniques represent a powerful method for rapidly and accurately detecting *P. pini* and contribute to the management and control of this harmful organism. Additionally, these techniques have advantages of cost-effectiveness, simple operation, and short detection times, and they are unaffected by site factors. Such innovative diagnostic methods are crucial for the early detection of invasive species and may enable effective mitigation strategies to be implemented before pathogens cause widespread damage.

## Figures and Tables

**Table 1:** Results of DNA detection in leaf samples based on PCR, RPA-LFD, and RPA-CRISPR/Cas12a.

Sample Number	Location ^a^	Detection of *P. pini*		
		PCR	RPA-LFD	RPA-CRISPR/Cas12a
1	JS, China	+	+	+
2	JS, China	−	+	+
3	JS, China	+	+	+
4	JS, China	−	+	+
5	JS, China	−	+	+
6	JS, China	+	+	+
7	JS, China	+	+	+
8	JS, China	+	+	+
9	JS, China	−	+	+
10	JS, China	+	+	+
11	JS, China	−	+	+
12	JS, China	+	+	+
13	JS, China	+	+	+
14	JS, China	−	+	+
15	JS, China	+	+	+
P1 ^b^	JS, China	+	+	+
P2	JS, China	+	+	+
P3	JS, China	+	+	+
NC1 ^c^	JS, China	−	−	−
NC2	JS, China	−	−	−
NC3	JS, China	−	−	−

^a^: province abbreviations: JS, Jiangsu; ^b^: P, positive control; ^c^: NC, negative control; “+”, the test result is positive; “−”, the test result is negative.

## Data Availability

The original contributions presented in this study are included in the article/Supplementary Materials. Further inquiries can be directed to the corresponding author.

## Supplementary Materials

The following supporting information can be downloaded at: https://www.mdpi.com/article/10.3390/microorganisms13040863/s1. Figure S1: Sequence comparison of sequence Ppini_05588 with *Phytophthora citricola*, *Phytophthora plurivora*. A: result of sequence Ppini_05588 with *P. plurivora*; B: result of sequence Ppini_05588 with *Phytophthora citricola*; “*”: two sequences compared to the same base; Figure S2: Sequence of the Ppini_05588 gene of *Phytophthora pini*. The nucleotides targeted by the forward (Ppini_05588RPA-F) and reverse (Ppini_05588RPA-R) primers, and the crRNA sequence are shown below the respective arrows. Arrows indicate the direction of amplification; Table S1: Information and results of Crispr-Cas12a detection on *Phytophthora*, other oomycete isolates, and fungal isolates utilized in this investigation. Table S2: The combinations of different concentrations of CrRNA and ssDNA reporter.
