# Supplementary figures and images for: Establishment of a Rapid Detection Technique Based on RPA-LFD and RPA-CRISPR/Cas12a on Phytophthora pini
Source: Microorganisms. 2025 Apr 10;13(4):863. doi: 10.3390/microorganisms13040863 (PMC12029582; doi:10.3390/microorganisms13040863)

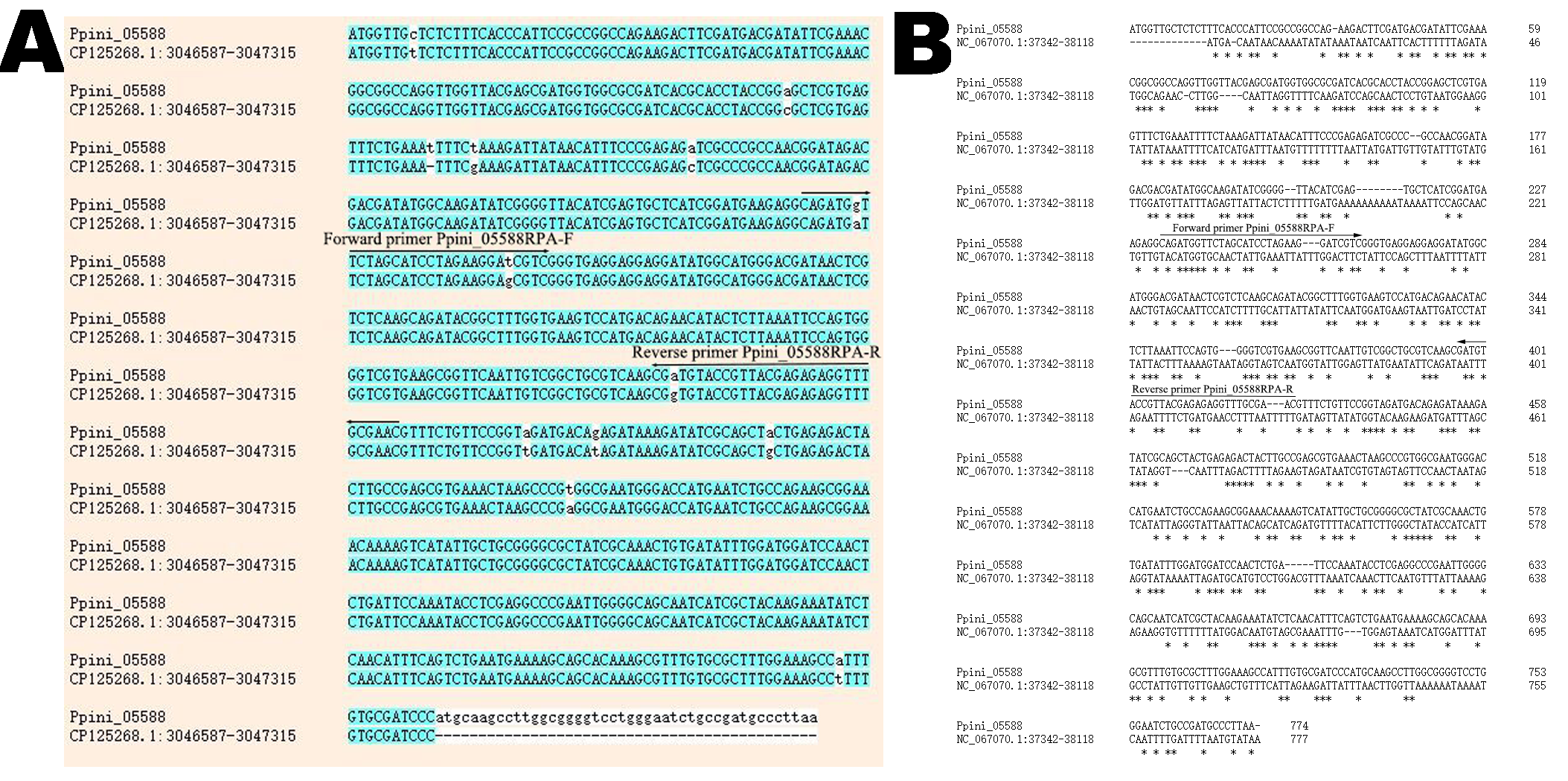

Supplement: Supplementary file 1 [file microorganisms-13-00863-s001.zip › Figure S1.tif]

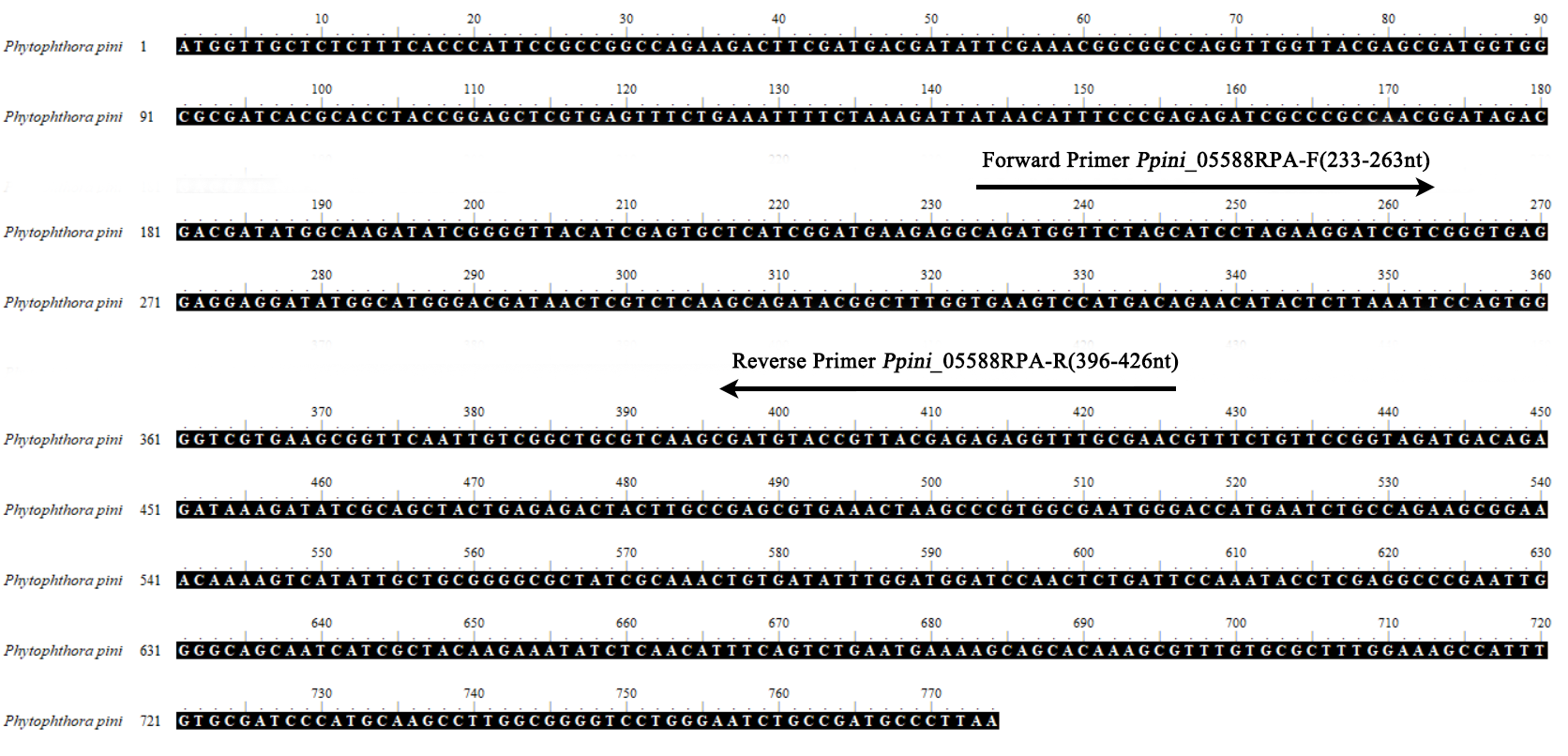

Supplement: Supplementary file 1 [file microorganisms-13-00863-s001.zip › Figure S2.tif]
